# Formative research on a post-discharge monitoring program using SQ-LNS to reduce acute malnutrition relapse risk in Mali : a qualitative study

**DOI:** 10.1186/s12889-026-28378-x

**Published:** 2026-07-03

**Authors:** Grace Heymsfield, Suvi T. Kangas, Bernardette Cichon, Issa Niamanto Coulibaly, Niele Hawa Diarra, Koniba Diassana, Alhousseyni Haidara, Bareye Ouologuem, Samba Diarra, Stephen Kodish

**Affiliations:** 1 International Rescue Committee, New York, NY USA; 2 International Rescue Committee, Brussels, Belgium; 3 International Rescue Committee, London, UK; 4 International Rescue Committee, Bamako, Mali; 5 Sub-division of Nutrition, Ministry of Health, Bamako, Mali; 6 University of Sciences, Techniques and Technologies of Bamako, Bamako, Mali; 7 Penn State University, Penn State, University Park, PA USA

**Keywords:** Mali, Formative research, Small-quantity lipid-based nutrient supplement (SQ-LNS), Severe acute malnutrition (SAM), Feasibility, Acceptability

## Abstract

**Introduction:**

Up to 75% of children treated for severe acute malnutrition (SAM) relapse within six months of recovery, yet evidence on post-discharge interventions remains limited. We conducted a formative assessment to understand how small-quantity lipid-based nutrient supplements (SQ-LNS) could be integrated in post-discharge care to prevent relapse.

**Objective:**

This study aimed to inform whether and how a program integrating SQ-LNS into post-discharge monitoring could be implemented in Mali. Specifically, we aimed to: 1) identify barriers and enablers to post-discharge monitoring, 2) develop one or more post-discharge models based on recommendations, 3) understand the feasibility of these model(s), 4) describe acceptability of the model(s) and product, and 5) propose a final model to be piloted at larger scale.

**Methods:**

This formative study comprised two iterative phases conducted between September 2024 and May 2025. In exploratory Phase One, we conducted semi-structured interviews with 12 caregivers of children with SAM and eight treatment providers, in triangulation with 13 focus groups among caregivers, health care workers (HCW’s), and supervisors. Themes included current practices, anticipated challenges, and service delivery preferences. Findings informed a confirmatory Phase Two involving four direct observations of three delivery models implemented at four sites and 13 interviews with similar participants. Textual data were coded and thematically analyzed using Dedoose software.

**Results:**

Although national guidelines recommend routine post-discharge monitoring for SAM children, it was rarely practiced due to limited awareness among HCW’s. Both caregivers and HCW’s supported the idea of post-SAM monitoring, preferring on-site over home visits for feasibility. Caregivers valued growth monitoring, interacting with HCW’s, and nutritional supplementation. While unfamiliar with SQ-LNS, caregivers viewed it positively based on their experience with therapeutic foods. Three delivery models were tested: (1) weekly visits for one month transitioning to fortnightly thereafter, (2) fortnightly visits and program days, and (3) fortnightly visits for caregivers and weekly program days at the health site. Model three proved most feasible for caregivers and providers.

**Conclusion:**

This formative research informed a program design that aligns with caregiver preferences and health system capacity, requiring minimal external support. Fortnightly post-discharge monitoring paired with SQ-LNS supplementation at treatment sites is recommended.

**Supplementary Information:**

The online version contains supplementary material available at 10.1186/s12889-026-28378-x.

## Background

Acute malnutrition, a form of undernutrition, is a biological coping mechanism to disease, infections, or inadequate dietary intake characterized by a rapid deterioration in nutritional status. According to cross sectional surveys, an estimated 45 million children suffer from acute malnutrition (AM) at any given time – a figure that underestimates the true burden of AM, as incidence is not accounted for [1, 2].

Over nine million children under five years were treated for severe acute malnutrition (SAM) in 2024 globally [3]. Outpatient therapeutic feeding programs use weight-based dosage of ready-to-use therapeutic foods (RUTF) to provide the full nutritional needs of children with SAM, with one or more sachets corresponding to 150–185 kilocalories per kilogram of the child’s weight given to the caregiver to administer at home [4–6]. In addition to RUTF, these programs provide systematic growth monitoring of height, weight, mid-upper arm circumference (MUAC), health checkups, and contact with a health care worker (HCW) at a health site typically on a weekly basis [6].

Most children treated for SAM are declared recovered within two to three months from the start of treatment [7, 8]. However, relapse to AM has been shown to affect many children who have been declared recovered [9–14] ranging from 21.9% to 63.1% within six months in a recent three-country study [11] and up to 75.8% according to a meta-analysis [15]. Higher weight-for-height z-score (WHZ), MUAC, and weight-for-age z-score (WAZ) at discharge are associated with lower risk of SAM relapse, with some studies documenting higher frequency of illness episodes per month of follow-up and severe food insecurity post-discharge as risk factors [10, 11, 15, 16].

Evidence on post-discharge interventions for SAM is limited across the globe, with the majority of evidence available for inpatient children [17]. The 2023 World Health Organization (WHO) guidelines provided two low-certainty evidence statements for post-SAM treatment interventions: psychosocial stimulation and cash transfers [6]. National Community-based Management of Acute Malnutrition (CMAM) guidance in certain high-burden countries stipulates post-discharge monitoring of children including health check-ups, growth monitoring, and health counseling for up to three months without therapeutic foods, or direct enrollment to the supplementation program for moderate acute malnutrition (MAM) with one daily sachet of ready-to-use supplementary foods (RUSF) for three months [18–21]. The effect of this monitoring on reducing relapse has not been documented.

Within the family of lipid-based nutrient supplements (LNS), which includes large quantity RUTF and RUSF, small-quantity LNS (SQ-LNS) were initially developed to address the micronutrient deficiencies of children 6–23 months of age in contexts where access to optimal complementary foods was poor [22]. These products are packaged in 20-gram individual sachets and provide key micronutrients, essential fatty acids and protein for young children in a sweetened paste (typically peanut-based) providing around 110 kilocalories/ day [23]. They are meant to be either consumed directly from the sachet by the child or mixed with complementary foods [22]. Among children 6 to 23 months of age residing in low-income settings, SQ-LNS have been shown to be effective in reducing mortality [24], severe wasting [25], severe stunting [25], iron deficiency anaemia [26], and developmental delay [27]. Secondary analyses of a longitudinal study of children under two years enrolled in integrated wasting prevention and screening program in Burkina Faso and Mali found that children who had received SQ-LNS who had also been enrolled in malnutrition treatment had 57–67% lower risk of relapse [14].

Given that relapse is associated with increased risk of adverse outcomes for children—including higher morbidity and mortality—greater opportunity costs for caregivers and added strain on already resource-constrained health systems, identifying strategies to improve post-SAM growth and prevent relapse risk is needed to improve outcomes for children, caregivers, and the broader health system [28]. Building on the potential of SQ-LNS to reduce relapse [14], we hypothesized that a post-discharge monitoring program that includes SQ-LNS supplementation, health checks and sensitization could reduce relapse by improving diet, reducing illness and improving initial treatment [29]. The objective of this formative research was to inform such a program in Mali’s Kati Health District. Specifically, we aimed to: (1) identify barriers and enablers to post-discharge monitoring, (2) develop one or more post-discharge models based on recommendations, (3) understand the feasibility of one or more post-discharge delivery models, (4) describe acceptability of the program and product, and (5) propose a final model to then be piloted.

## Methods

### Study setting

Data was collected from October 2024 to May 2025 in the Kati health district in Mali’s Koulikoro region, a large district with urban, peri-urban and rural areas proximal to Bamako. Mali has faced persistently high acute malnutrition rates of around 10% for decades, with alarming projections showing 1.6 million children aged 6–59 months expected to suffer from acute malnutrition in 2025 - an 11% increase from the previous year [30]. In Kati specifically, recent Integrated Food Security Classification Framework (IPC) data projected 92,727 children would suffer from AM from June 2024 to May 2025, including 18,831 severe cases [30]. Regional statistics for common morbidities are similar to the national average, as children suffering from diarrhoea, fever, and acute respiratory infection (ARI) were 14%, 18%, and 13% respectively according to the most recent SMART survey, and care-seeking practices for these morbidities ranging from 40 to 55% [31, 32]. Phase One data collection coincided with the main harvest season and Phase Two during the off-season harvest, when SAM admissions are expected to be lower than the yearly average.

At the time of the study, the district’s malnutrition treatment infrastructure included 44 functional Community Health Centers (CSCOM for “*Centre de Santé Communautaire*”) with Outpatient Therapeutic Programs and a 24-bed inpatient care unit. In 2015, the government modified its primary health care policy to include outpatient treatment of uncomplicated SAM within the package of integrated Community Case Management (iCCM) activities that community health workers (CHW’s) are expected to carry out in their villages at functional secondary sites supervised by staff at the CSCOM [33]. Of the approximately 100 secondary sites in Kati managed by CHW’s at the time of the study, only 38 were implementing SAM treatment.

In Mali, children with uncomplicated SAM are treated with RUTF until achieving non-AM anthropometry (WHZ ≥ -1.5, MUAC ≥ 125 mm and no edema for two consecutive weeks). Treatment in secondary sites is simplified to children with a MUAC < 115 mm and no complications. The national protocol of Mali specifies post-discharge monitoring for SAM treatment. According to the 2022 national protocol, children should be followed up post-SAM discharge in their village with growth monitoring, health counseling, and referrals as needed for a duration of three months by the CHW, community nutrition support group, or Community Health Volunteers (CHV’s) (once a week for the first month and once every two weeks during months two and three). In the absence of decentralized health workers, this monitoring program is implemented at the nearest health facility [21]. Comparatively, according to the 2011 and 2017 national protocols, at discharge children treated for SAM should be enrolled to the MAM supplementation program for three months, where they receive a dose of one daily sachet of RUSF for weekly then fortnightly visits, or three months of follow-up without nutritional support if the MAM supplementation program is not functional [20, 34].

### Study design, sampling, and data collection procedures

This formative research was conducted in two iterative phases using multiple methods: first an “exploratory” phase and a second “confirmatory” phase whereby qualitative data were collected from caregivers and HCW’s. We used triangulation of caregiver and HCW preferences and recommendations to develop and then observe different models of post-discharge monitoring activities. We followed the consolidated criteria for reporting qualitative studies (COREQ) in reporting the findings (Supplementary File 1: COREQ) [35].

A two-tiered, theoretical, and criterion-based purposive sampling strategy developed to enhance representativeness of the health district was used to recruit participants. A reasoned choice was made such that caregivers and HCW’s were recruited from six of the 44 health areas of the health district, each composed of one CSCOM and their associated secondary site(s), with equal representation across each milieu of the district (rural, semi-urban, urban). Additional considerations included representativeness across SAM caseload (low, medium, and high compared to the district average) and distance to Bamako (for accessibility by the research team – proximal (< 30 min), short-distance (30 min to less than one hour), and longer-distance (one hour or more)). Within these six health areas, we sampled the number of participants needed to reach data saturation among key themes [36].

#### Exploratory phase (Phase One)

Phase One used semi-structured interviews (SSI’s) (*n* = 20) with caregivers (*n* = 12) and implementing HCW’s (*n* = 8) to understand supply and demand-side factors that should be considered by a post-discharge monitoring program that included SQ-LNS. Subsequently, focus group discussions (FGD’s) (*n* = 13) with caregivers (*n* = 5) and implementing HCW’s (*n* = 6) were used to develop strategies to overcome expected challenges by a post-discharge monitoring program, including picture ranking exercises on the program location, frequency, and services. Based on responses from caregivers and implementing HCW’s, FGD’s were then held with supervisory HCW’s (*n* = 2) to build consensus on implementation models to pilot. SSI and FGD guides were semi-structured and organized by research themes, including visual aids and ranking exercises, internally tested and pilot tested in a catchment area of Kati excluded from the formative research sampling framework [37–39].

SSI’s and FGD’s were conducted by a team of four trained qualitative investigators (two women and two men) recruited from the Bamako context under the direct supervision of a qualitative research expert from the University of Sciences, Techniques and Technologies of Bamako (SD) and a Senior Research Manager (KD). The investigators were fluent in Bambara and worked in pairs (each team included one male and one female investigator). Back translation exercises in French and Bambara were included as part of investigator recruitment and training.

#### Confirmatory phase (Phase Two)

A validation exercise was held with the Ministry of Health prior to introduction of program activities in Phase Two. Four of the six health areas were purposively sampled based on their representation of each milieu of the Kati context and expected program enrollments. During Phase Two, we conducted direct observations (*n* = 4) of post-discharge monitoring with health check-ups, behavior change communication, anthropometric monitoring, and SQ-LNS distribution after one month of program implementation. The pilot activities were implemented from February 2025 to May 2025 (16 weeks).

Three observers with malnutrition treatment and clinical experience (KD, INC, HD) used a semi-structured checklist assessing fidelity to the program protocol [40]. Categories that were assessed during each observation included reception and triage, anthropometric monitoring, clinical examinations, SQ-LNS distribution and counseling, and completion of program tools (participant card, program registry, and individual monitoring cards). In addition, we interviewed both caregivers (*n* = 9) and implementing HCW’s (*n* = 4) at each program site to triangulate observational data.

### Data analysis

#### SSI’s and FGD’s

All SSI’s and FGD’s were audio recorded. Recordings were translated and transcribed verbatim from Bambara into written French by the research team, maintaining local terminology [41]. Transcripts were reviewed in Word for completeness and clarity on an iterative basis, and a sub-set (5%) were transcribed by a second researcher (SD) and compared to the original for completeness.

Dedoose software was used for data management and coding [42]. An initial codebook with 42 codes was developed based on semi-structured guide questions that were developed a priori and reflected the domains explored in both SSI's and FGD's. Prior to coding, a set of guidelines were established among three analysts, including inter-coder reliability agreement of *≥* 0.80 [43, 44]. During the coding process, eight additional codes were added as a result of emergent themes (Supplementary File 2: Codebook.)

#### Direct observation data

Semi-structured observation data (e.g., descriptions of program activities and implementation quality) were recorded with standardized forms. Descriptive field notes were then audio recorded by the investigator at the end of each observation, then transcribed into written French and translated to English. The research team compiled these field notes and transcriptions in Dedoose software and analyzed key themes relevant to the research questions.

After thematic coding of text data, results were summarized by research aim and shared with co-investigators to validate interpretation, in written summary and select interpretations via calls (SK, SK, GH, INC, KD). We followed the consolidated criteria for reporting qualitative studies (COREQ) in reporting the findings (Supplementary File 1: COREQ) [35].

### Ethical approval

The study protocol was approved by the Institutional Review Board of the International Rescue Committee (H 1.00.080) and the Ethical Committee of the University of Sciences, Techniques and Technologies of Bamako (N.2024/211/USTTB). The study was conducted in accordance with the Declaration of Helsinki. Informed oral consent was obtained from participants.

## Results

### Barriers and enablers to post-discharge monitoring

#### Post-discharge monitoring is not offered

Children with SAM do not receive systematic follow-up care after discharge, neither at health sites nor at home. When asked about post-discharge monitoring as defined in the 2022 national protocol, HCW’s either were not aware of this stipulation or recalled their training with the 2017 national protocol, which states SAM children at discharge as fully recovered (WHZ > -1.5 calculated from target weight at enrollment) are enrolled to nutritional supplementation with RUSF for 3 months if the product is available [20], or trainings even prior to 2017 based on the 2011 protocol [34]. Per the 2011 and 2017 national protocol, in areas where RUSF is available, children do appear to be enrolled at discharge from SAM.

***“****At the moment*,* these children don’t benefit from any follow-up*,* which is due to the lack of MAM stock. If the children have been cured from SAM*,* they have to follow the MAM treatment*,* and we don’t have the PlumpySup (RUSF). If we had the MAM stock*,* we’d follow them up by going to them or calling on them.”* – SSI with a HCW, Phase One

Post-discharge monitoring in the absence of RUSF does not appear to be in place, and most sites did not have RUSF available at the time of the study. CHV’s are tasked with home follow up and screening activities when these activities are conducted, but these are rare and linked to screening and community outreach campaigns (vaccination, vitamin A, and/or deworming). Some HCW’s understood community screening activities (which are generally preventative in nature) as part of general child growth monitoring, including for post-SAM children. Community campaigns are typically incentivized by a per-diem to implement the activity.

#### Existing perception of RUTF and SAM treatment is generally positive

Among caregivers whose children had already completed SAM treatment, RUTF was favorably perceived as being the reason that their child gained weight, got stronger, regained energy, got sick less often, and/or could play again, compared to when they were severely malnourished and sick. RUTF is perceived by caregivers as a medicine for children and not food. Knowing that it is a medicine helps them explain to family members and community members that it is only used for the child when/ if receiving community pressure to share. Nevertheless, caregivers mentioned that there are a few instances where treatment products are sold per observations in the market.

Some side effects (diarrhea, blood in stools) were associated with RUTF by some caregivers, especially at the start of treatment. RUTF stockouts contribute to distrust in the program and major implementation disruption.

***“****The only difficulty can be stock-outs. That’s the difficulty we experience because you leave home sometimes and you’ll find that the RUTF is out of stock. You go back. Apart from this difficulty*,* if you go and find the RUTF there anyway*,* I don’t see any difficulty.”* – SSI with a caregiver, Phase One

In addition to RUTF stockouts, HCW’s expressed frustration with resource constraints to ensure continuity of care during SAM treatment, such as lack of routine antibiotics or faulty anthropometric equipment. HCW’s felt this contributed to feelings of disappointment from caregivers, and they did not want to be in a similar situation if offering post-discharge monitoring with SQ-LNS or another product or medicine.

#### Caregivers would be incentivized to participate in post-discharge monitoring with trusted HCW’s

The perceived burden and risks of malnutrition (not necessarily distinguishing relapse from the initial malnutrition episode) is high enough that caregivers would feel the urgency to participate in a program if it was intended to prevent malnutrition. Some kind of “*commission*” or service should be part of the routine program if it requires follow up visits or actions by the caregiver.

Where MAM supplementation with RUSF is offered to post-SAM children, it is anecdotally well attended. Preserving the relationship with trusted “*big*” HCW’s (who oversaw their child’s SAM treatment) is important to most caregivers “*because they’re the ones who treated him and brought him out of malnutrition*,” rather than getting transferred over to a “*small*” health agent like a CHV in their community.

When asked, no caregivers had received a home visit outside of ad-hoc community-based screening or vaccination campaigns. If post-discharge monitoring were offered at the caregiver’s home or village instead of the health site, they’d still like the HCW responsible for SAM treatment to assure the visits.

Other elements that caregivers would like to be part of post-discharge care included growth monitoring and counseling and some kind of commission or service (food, therapeutic food, or medicine).

### Recommendations for post-discharge monitoring with SQ-LNS

#### Post-discharge monitoring should be offered at health sites weekly or fortnightly

While some advantages of home delivery of services were mentioned, at the time of the study there were no existing programs in Kati where the other desired program components (growth monitoring, health checks) were implemented outside of the health site (CSCOM and/or secondary sites). When asked to think of such a program (home or community service delivery), caregivers conceded that HCW’s time was more important than their own and that it was their responsibility to attend the site for the good of their child, as HCW’s are experts.

*“For me*,* it’s better to come according to the deadlines(schedule) they give us at the health center (CSCOM). Because you’ll find that they need certain materials for checking the child*,* which can be cumbersome if they have to move around with them. It can be a particular inconvenience for them. If we even come to the center*,* once we’ve finished we go home. That’s what I think.”* – SSI with a caregiver, Phase Two

SSI’s indicated and subsequent FGD’s confirmed that a post-discharge monitoring program in this context should be delivered at health sites because it (1) is a continuation of SAM treatment delivered at those sites and (2) includes medical components (screening, checkups, etc.). Caregivers worried that, if there was home delivery and SQ-LNS shortages, those who stayed at home would not be prioritized and might “*get left behind*” by the program. Some caregivers may not accept home visits or might question the quality of the product if it’s delivered at home – this was particularly emphasized by HCW’s as a likely constraint based on their experience with immunization programs. Furthermore, home service delivery was perceived as a new program implemented by an external partner that may go away if the project funding went away and would require additional per-diems to deliver.

Since SAM is a medical condition, post-SAM / relapse prevention was associated by caregivers with medical care too (especially if the SQ-LNS product is communicated as medicine, not food). Therefore, most saw post-discharge monitoring as a medical program that should happen at a health site by a HCW. HCW’s felt that continuing post-discharge care at the same site/ with the same person or team who oversaw SAM treatment was most feasible – unless a child was treated at the CSCOM but lived closer to a secondary site and could be transferred over to the CHW.

HCW’s at the CSCOM felt secondary sites could and should implement the program if they are equipped to deliver SAM treatment. The functionality of SAM treatment at secondary sites was low (due to lack of resources, training’s, RUTF supply), so HCW’s at CSCOM’s said a first step would be improving functionality of SAM treatment at those sites. Recommended modifications to the protocol in secondary sites included growth monitoring by MUAC and edema only as per the SAM treatment protocol (removing the stipulation of WHZ).

Regarding frequency, HCW’s and caregivers saw advantages and disadvantages of weekly or fortnightly delivery (referencing the SAM and MAM programs that function with these frequencies in most sites). Most felt monthly visits were too infrequent for monitoring the child, and too many SQ-LNS sachets for the caregiver to manage between visits. Strong preferences for weekly versus fortnightly visits were generally not expressed, though the cumulative caseload of a weekly model was cited as a potential feasibility concern. The main reason to recommend a weekly visit from HCW’s was so that caregivers wouldn’t “*waste*” or misuse the SQ-LNS and that they saw the advantages of regular checkpoints/ monitoring of the child. Considering that SAM treatment days were typically aligned with market days or other activities in the community, the day and time of service should be set by the site but generally followed the weekday and time of SAM treatment.

#### Three recommended models for implementation modified program frequency

By triangulating recommendations from caregivers and implementing HCW’s, three models for implementation at health sites were recommended for Phase Two: (1) a weekly then fortnightly visit according to the national protocol, and (2) a fortnightly visit for caregivers programmed at the health site either (a) every week or (b) every two weeks (Table 1). Children finishing SAM treatment were offered three months of participation to the post-discharge program, with children admitted on a rolling basis during the sixteen-week pilot period. A standard operating procedures (SOP) document was elaborated defining the program protocol for SQ-LNS distribution, health checkup procedures, and key messages.

**Table 1:** Description of sites and models for piloting

	Site(s)	Milieu	Model
Health Area 1	CSCOM 1	Urban	A: Weekly visits for one month then fortnightly thereafter
Health Area 2	CSCOM 2	Peri-urban	B.1: Visits every two weeks for caregivers; program scheduled once a week at the health site
	Secondary Site 1		
Health Area 3	CSCOM 3	Rural	B.2: Visits every two weeks for caregivers; program scheduled once every two weeks at the health site
	Secondary Site 2		
Health Area 4	CSCOM 4	Peri-urban	

Program components included SQ-LNS supplementation corresponding to one sachet per day, anthropometric monitoring at every visit (weight, MUAC, and edema every visit, and height monthly at CSCOMs), morbidity recall, vaccination checks, and nutrition counseling. The program was implemented by routine HCW’s responsible for SAM treatment.

### Feasibility of observed delivery models

#### Feasibility of the models was reported and observed

HCW’s reported that the program was feasible to implement. The training they received for SAM treatment and the post-discharge monitoring activity increased their confidence in implementing both. Mirroring as many components as possible from the SAM treatment program (i.e. similar registries, individual program cards) further enhanced feasibility. Fortnightly visits were suggested as more feasible for both caregivers and providers than weekly or monthly visits (or a phased weekly-to-fortnightly schedule).HCW’s viewed post-discharge monitoring as a natural extension of their role in SAM care, given the relationships they had already established with caregivers and children. Table 2 summarizes feasibility considerations and observations.

**Table 2:** Feasibility considerations and observations, Phase Two

	Weekly	Fortnightly	Monthly (not observed)
Cumulative caseload of children seen at the site	Highest	➔	Lowest
SQ-LNS sachets to manage for caregivers	Easiest	➔	Most difficult
Number of health checks provided for children	Most frequent	➔	Least frequent
Time to travel for caregivers to the site	Most burdensome	➔	Least burdensome

The basic program components, as reported, were well understood, but some specific nuances were not and required correction, including:

- Respect of the transition visit for the weekly then fortnightly rhythm (Model A), which was observed as being one week too early or late,
- Immediate enrollment at discharge if delay in program offer (Model B.2) – children discharged from SAM treatment in an “off” week were asked to come back one week later for SQ-LNS,
- Monthly height measurement and re-calculation of the WHZ based on this height – height was often taken at enrollment only and not updated monthly.

Additional challenges that persisted during the confirmatory phase for SAM treatment included lack of routine antibiotics, deworming, and sufficient time for nutritional education and thorough morbidity screening.

Day and time of service was set by the site but generally the same as the SAM treatment day (which is set to consider market days and other community preferences). Over the sixteen weeks of observation, the program was generally functional with some key areas for quality improvement (behavior change communication (BCC), screening for morbidities). The same staff (on the same day and generally the same time) implemented SAM treatment and post-SAM monitoring, with children seen in the order of their arrival to the site whether enrolled to the SAM, MAM, or post-SAM programs. While introduced to two secondary sites, no program activities were observed during the sixteen weeks of observation due to no discharges from SAM treatment in that time. In theory HCW’s felt that the program could be feasible in a secondary site but pointed to the effective non-functionality of SAM treatment as the limitation.

### Program and product acceptability

#### Caregivers expressed acceptability of the product and monitoring

Seeing their child grow, play, gain weight and remain healthy motivated caregivers to attend visits and administer the SQ-LNS according to the recommended dose. Caregivers attributed positive changes in their child- including an upward trend in height and weight - to SQ-LNS as part of the child’s recovery from SAM. The program was explained to caregivers as preventing another malnutrition episode, which they said was what made it desirable to them – recalling the gravity of their child’s previous situation.

However, the SQ-LNS product was perceived as small, compared to RUTF. Caregivers reported children request multiple sachets and that they sometimes conceded.

***“****It’s not much*,* this one (my child) cries. As it’s recommended one sachet*,* that’s why we let it*,* otherwise it’s not much… Because when we had the other one (RUTF) even if we were going into town*,* you bring a sachet with you without being worried. Once he’s hungry*,* you give it to him and you come back without any problem. But with this product*,* it’s not even worth bringing it along (Laughs).”* – SSI with a caregiver, Phase Two.

Caregivers varied in their preparation methods: some said they gave SQ-LNS alone while others reported mixing it with porridge. When interviewing or following up with caregivers who had missed visits or discontinued the program, absence or discontinuation was reported as being related to family events or temporary migration, rather than dissatisfaction with the product or the services.

No consistent/ strong recommendations emerged from caregivers regarding changes to program location. For visit frequency, caregivers were generally deferred to the frequency assigned to them by HCW’s. Some caregivers enrolled at sites with fortnightly frequency voiced hesitancy about more frequent movements (weekly), but this was not a consistent preference across participants.

#### A communication strategy should promote appropriate use and differentiate SQ-LNS from RUTF

Based on observations, the general messages shared about SQ-LNS by HCW’s were basic and typically limited to correct dose per day and hygiene messages. HCW’s suggested that a communication strategy to improve the likelihood of SQ-LNS compliance in this setting have two overarching goals: (1) promote the appropriate use of the supplement, and (2) distinguish it from RUTF already being used in the area as part of SAM treatment. The primary suggested communication channels were interpersonal involving HCW’s at clinics and other caregivers who had previous experiences with SQ-LNS, as well as awareness campaigns at community level. Time was cited as a constraint for counseling, in the event that the HCW had several children waiting in queue. Table 3 summarizes marketing for the product based on Phase One and Phase Two recommendations and observations.

**Table 3:** SQ-LNS distribution and communication strategy

Principle	Summary of Phase One and Phase Two
Provider and location	• SQ-LNS should be distributed at the health site by a trusted HCW preferably on the same day caregivers had previously attended SAM treatment
	• If delivered at home instead of a health site, some caregivers expressed concern about doubting the quality of the product
Product positioning	• Caregivers associated SQ-LNS with RUTF or RUSF, but described it as *“small”*
	• Suggested names for branding SQ-LNS included: “*Fangadèguèni* - the peanut paste that give strength”, “*the small RUTF*”, “*RUTF’s little sister*,” and “*the green that follows the red*” (referring to the color coding of RUTF and SQ-LNS packages)
	• SQ-LNS was perceived to maybe be “*stronger*” than RUTF, because it was small and the child only needed one sachet per day instead of multiple
Service delivery	• Because SQ-LNS is perceived as a medical product from the health site, caregivers would not pay for it (and have been sensitized to RUTF being forbidden to sell while participating in SAM treatment)
	• Caregivers said the time and energy associated with the visit if SQ-LNS was provided was worth anything for the child’s health, but could be a burden insurmountable by family obligations, income generating activities, or emergencies
Promotion and Awareness campaigns	• HCW’s (“*big*” health workers responsible for the child’s SAM treatment) are trusted for the SQ-LNS distribution and inherently promotion, because they have already seen the child through their SAM episode (vs. CHV’s or “*small*” health workers): during visits, or in group settings at the treatment site.
	• Peers are a trusted source of information for utilization of RUTF, including uses not advised by HCW’s (boiling RUTF, mixing it with family foods, and storing opened sachets for extended periods).

## Discussion

This study aimed to investigate if and how a post-SAM monitoring program proposing SQ-LNS could be integrated into the existing health system. Triangulating caregiver insights with implementing and supervisory HCW perspectives in two iterative phases allowed us to develop and then observe three models of delivery. We learned and then confirmed that post-discharge monitoring should maintain continuity by keeping caregivers in contact with the same HCW’s who provided treatment services. The intervention should include monitoring of the child’s growth and nutrition education, comprehensive screening procedures for acute malnutrition, with some kind of nutritional support. Fortnightly visits were most feasible for the health system and caregivers, allowing frequent monitoring while also avoiding unsustainable cumulative caseloads for HCW’s.

Our findings suggest that providing a nutritional supplement is critical for the functionality of both SAM treatment and post-discharge monitoring. While most HCW’s were not aware of the provision of post-discharge monitoring in the national protocol, they were skeptical of how one would work without an incentive to encourage attendance. This is consistent with studies in West Africa, where SQ-LNS acted as an incentive to attend screening, increasing coverage by 23–40% compared to offering the same services without SQ-LNS [45, 46].

We observed SAM treatment protocol violations that may contribute to relapse, including exit before reaching the recommended WHO criteria for discharge or lapses in the quality of treatment components, especially during RUTF stockouts [6, 47]. This is concerning, as successful outcomes for children enrolled in post-discharge monitoring may depend on the quality of initial treatment. In Nepal, for example, a study found that children treated for SAM who failed to reach WHO-recommended discharge criteria (i.e. failed to achieve WHZ ≥-2 when presenting a WHZ<-3 at admission and a MUAC ≥ 125 mm when presenting a MUAC < 115 mm at admission) had a much higher risk of relapse [48]. Our work highlights that post-discharge programs should ideally strengthen the continuity of care across all phases – from screening and treatment through post-discharge monitoring - to ensure that children are detected early, receive home follow-up visits to reduce defaulting, and achieve full anthropometric recovery prior to discharge [16].

While acceptability of RUTF and SQ-LNS have been widely demonstrated in prevention and treatment programs respectively, in this post-SAM population, SQ-LNS acceptability was further enhanced by the caregiver’s positive experience with RUTF and seeing their child successfully complete treatment and become healthier [49–51]. SQ-LNS was referred to as “*the little RUTF*” or “*RUTF’s little sister*,” likening the two products while also acknowledging their different sizes. Key messages should aim to further distinguish the two products from each other (as well as RUSF where it is used for MAM supplementation) and preserve their different intended uses, especially where measurement error or protocol deviations may result in the wrong nutritional product being provided to children with SAM, MAM, or post-treatment [45]. As SQ-LNS is approximately one-fifth the size of RUTF and is meant to be supplementary to regular food rather than a meal substitute, acceptability over time will be important to monitor, through both program adherence and longitudinal product use and acceptability. HCW’s as well as peers were trusted promoters for the product, implying that communication strategies should ensure caregivers are also trained on the appropriate use of SQ-LNS.

A core question of our research was who should provide post-discharge services. Both caregivers and HCW’s thought services should come from the same SAM treatment providers and not CHV’s. Caregivers indicated they trusted HCW’s competencies, saying it was important that a HCW should be able to respond to their questions and that a CHV would be unlikely to. This finding aligns with evidence showing that trust and training are interconnected – caregivers perceive health workers (paid CHW’s or volunteer CHV’s) providing community-based services to mothers and infants as more trustworthy when they are educated, knowledgeable, and have received adequate training [52]. Our work underscores the need to train and equip CHV’s and CHW’s to be seen as health experts in their community, to build trust in decentralized services which could increase coverage and reduce caregiver opportunity costs.

Related to the question on the optimal provider was the question on where the services should be provided: at the treatment site or through home visits. Both caregivers and HCW’s thought services should be provided at the same sites at treatment. Different advantages and disadvantages of facility versus decentralized community delivery of SQ-LNS have been reported, albeit as part of general malnutrition prevention programs for young children and not for a higher-risk, targeted population such as children recovering from SAM [45, 46].

Facility-based services offer the advantage of more comprehensive growth monitoring, as height and weight scales are readily available at health facilities, allowing for screening by all anthropometric indices used to define SAM, rather than MUAC and edema only [6, 45]. This is particularly important given that concordance between WHZ versus MUAC and edema is generally poor, especially for older children, and a MUAC and edema-based program in a community setting may “miss” relapsed children [45–47, 53]. However, facility-based services present challenges. Increased opportunity costs to caregivers—including time spent traveling and waiting at health sites—may contribute to lower adherence to service attendance [45]. Additionally, the effectiveness of behavior change communication may be compromised in either setting: HCW workload in facility settings may limit time for adequate counseling, while community-based services may suffer from variable quality depending on CHV training and supervision [54–56].

For post-discharge monitoring with SQ-LNS, fortnightly visits at the health site were seen as a good compromise between feasibility and effectiveness. Given the consistent feasibility and acceptability concerns expressed by both caregivers and HCW’s about post-discharge monitoring through home visits, we did not implement a model of the program outside of health sites in the pilot phase. While SAM treatment is typically provided on a weekly basis, questions on the opportunity costs of this frequency for caregivers as well as the cumulative caseload raised doubts on the sustainability and need of this visit frequency post-SAM. Transitioning from weekly to fortnightly visits was observed to be confusing for HCW’s, even when implemented at small-scale for an enrollment period of only three months – leaving questions as to how feasible this would be to monitor for a larger caseload of children over time. Generally, relapse is recommended to be monitored for 6 months [57]. The current study only provided the intervention for 3 months to align with national protocol and due to study time constraints, but a scaled program might be advised to apply a 6 month follow-up, requiring careful monitoring of feasibility due to increased caseload.

The feasibility of comprehensive anthropometric assessment during post-discharge monitoring also warrants consideration and monitoring over time. Studies in pediatric settings have shown that implementation of standard height measurement methodology is not always feasible due to environmental obstacles or staff non-compliance, whereas the post-discharge monitoring activities introduced required height on a monthly basis [58, 59]. Novel interventions should ultimately balance HCW workload and the frequency of contact for determining relapse to ensure highest quality of care [60]. The proposed model fits within global guidance for relapse, which recommends monitoring children for at least six months with non-specified regularity [57].

This study had several strengths. First, the iterative nature of our work enabled us to use Phase One findings to inform the design of three implementation models for Phase Two. We used various methods including semi-structured interviews, focus group discussions with validation exercises, and direct observations of program activities, which gave the research team a rich understanding of intervention design elements as reported and observed. Second, our target population was well-defined: HCW’s and caregivers were recruited based on their participation in the SAM treatment program, ensuring that recommendations for post-discharge monitoring were informed by current and recent experiences. Third, the observation period during Phase Two was sixteen weeks, which allowed us to understand program feasibility over time, rather than just cross-sectionally.

Our study had several limitations. First is that both phases were conducted outside of the peak AM season, during the period of lowest SAM admissions. Consequently, many questions and observations about feasibility or cumulative caseload were hypothetical in nature. Additionally, MAM programming was not functional at the time of our observations. Thus, the observed case load was lower than it would be if SAM, MAM, and post-SAM children were all being seen at the site on the same day. It will be important not to overwhelm the system when introducing post-discharge monitoring which could lead to lower quality of services [60]. Over time, further spacing visits, modifying triage, or changing service provision days may be needed.

Second, we were not able to observe program activities in secondary sites due to low admissions to SAM treatment, which translated to no enrolments in the post-discharge monitoring in these areas during the sixteen-week pilot. While this speaks in part to the low functionality of SAM treatment in these sites which could be strengthened- an important finding in and of itself- it is important to understand that feasibility of post-discharge monitoring when implemented by CHW’s would need further investigation. However, previous studies have demonstrated CHW capacity to implement SAM treatment [61, 62]. 

Third, in the exploratory Phase One, perceptions and impressions of SQ-LNS were limited to visual aspects and packaging only, not taste, as children should only consume RUTF during SAM treatment [6]. Considering the available evidence on SQ-LNS acceptability in young children [49–51], and the demonstrated acceptability to the RUTF product for children completing treatment in our study, we felt acceptability questions about the taste of the product at child level were not necessary. Instead, taste acceptability was explored according to caregivers of children enrolled to the post-discharge monitoring program during Phase Two.

## Conclusion

To our knowledge, this is the first formative research study to tailor a malnutrition prevention program including SQ-LNS for children discharged from SAM treatment. We found that post-discharge monitoring for children with SAM would be feasible and acceptable to both caregivers and HCW’s, particularly when a concrete incentive such as SQ-LNS is provided and when the service is offered by the same trusted HCW’s at the same locations where treatment happened. Fortnightly visits emerged as the preferred frequency in this setting, balancing adequate monitoring with caregiver convenience and HCW capacity. These findings suggest that post-discharge services should be integrated into existing SAM treatment platforms rather than developed as separate interventions. While this study demonstrated feasibility during a period of lower caseload, questions remain about optimal delivery models during peak malnutrition seasons. Future work should prioritize: (1) longitudinal evaluation across seasonal caseload variations and (2) effectiveness research quantifying impact on growth and relapse.

## Supplementary Information

Supplementary Material 1.

Supplementary Material 2.

## Data Availability

Qualitative tools are available on request. The data that support the findings of this study are available on request from the corresponding author. The data are not publicly available due to privacy or ethical restrictions.

## Abbreviations

AM: Acute Malnutrition
ARI: Acute respiratory infections
BCC: Behavior change communication
CHV: Community Health Volunteers
CHW: Community health worker
CMAM: Community-based management of acute malnutrition
COREQ: Consolidated criteria for reporting qualitative studies
CSCOM: Centre de Santé Communautaire (Community Health Centers)
FGDs: Focus group discussions
HCW: Health care worker
ICCM: Integrated Community Case Management
IPC: Integrated Food Security Classification Framework
IRB: Institutional Review Board
IRC: International Rescue Committee
LNS: Lipid-based nutrient supplements
MAM: Moderate Acute Malnutrition
MUAC: Mid-upper arm circumference
MOH: Ministry of Health
RUSF: Ready-to-use supplementary foods
RUTF: Ready-to-use therapeutic foods
SAM: Severe Acute Malnutrition
SMART: Standardized Monitoring and Assessment of Relief & Transitions
SOP: Standard operating procedures
SQ-LNS: Small-quantity lipid-based nutrient supplements
SSI: Semi-structured interview
USTTB: University of Sciences, Techniques and Technologies of Bamako, Mali
WAZ: Weight-for-age z-score
WHO: World Health Organization
WHZ: Weight-for-height z-score
