# Supplementary material for: Formative research on a post-discharge monitoring program using SQ-LNS to reduce acute malnutrition relapse risk in Mali : a qualitative study
Source: BMC Public Health. 2026 Jul 3;26:2296. doi: 10.1186/s12889-026-28378-x (PMC13445792; doi:10.1186/s12889-026-28378-x)
Supplement: Supplementary file 1 [file 12889_2026_28378_MOESM1_ESM.docx]

**Consolidated criteria for reporting qualitative research (COREQ): 32-item checklist**

| **Item number** | **Guide questions** | **Reported on** |
| --- | --- | --- |
| **Domain 1: Research team and reﬂexivity** | | |
| *Personal Characteristics* |  |  |
| 1. Interviewer/facilitator | Which author/s conducted the interview or focus group? | Data collection in Methods section. |
| 2. Credentials | What were the researcher’s credentials? E.g. PhD, MD | The four trained qualitative investigative research officers held Masters or advanced training in relevant fields. Dr. Samba Diarra is a PhD in health sociology, Dr. Koniba Diassana is general practitioner (MD), Dr. Issa Niamanto Coulibaly is MD and MPH, and Alhousseyni Haidara is a Statistician. |
| 3. Occupation | What was their occupation at the time of the study? | Qualitative investigative research officers were hired by the IRC as specific recruited staff for the research.  Dr. Samba Diarra was Professor and researcher, research fellow in the Department of Public Health and Specialties, coordinator of community engagement activities at the University Clinical Research Center, University of Science, Technology, and Technology, Bamako, Mali. (USTTB) and was hired as a consultant for the study.  Three full-time IRC research team members assisted data collection during direct observations (Research Coordinator, Senior Research Manager, Data Manager Analyst). |
| 4. Gender | Was the researcher male or female? | Both. Teams were composed in two teams of two (one male and one female) during Phase One, and one male investigator continued data collection in Phase Two. |
| 5. Experience and training | What experience or training did the researcher have? | Training in qualitative data collection and public health. |
| *Relationship with participants* | | |
| 6. Relationship established | Was a relationship established prior to study commencement? | No relationship was established prior to study commencement. HCW’s may have met the qualitative investigator retained for Phase Two during Phase One. |
| 7. Participant knowledge of the interviewer | What did the participants know about the researcher? e.g. personal goals, reasons for doing the research | Participants were aware of the primary purpose of the study (Data collection in Methods section). |
| 8. Interviewer characteristics | What characteristics were reported about the inter viewer/facilitator? e.g. Bias, assumptions, reasons and interests in the research topic | Interviewers had extensive experience in qualitative research (Data collection in Methods section). |
| **Domain 2: study design** |  |  |
| *Theoretical framework* |  |  |
| 9. Methodological orientation and Theory | What methodological orientation was stated to underpin the study? e.g. grounded theory, discourse analysis, ethnography, phenomenology, content analysis | Thematic analysis (Data analysis in Methods section). |
| *Participant selection* |  |  |
| 10. Sampling | How were participants selected? e.g. purposive, convenience, consecutive, snowball | Purposive sampling was employed (Participants in Methods section). |
| 11. Method of approach | How were participants approached? e.g. face-to-face, telephone, mail, email | **Phase One**  For SSI’s, HCW’s at treatment sites (CSCOMs and their associated secondary sites) were asked to identify caregivers who had a child currently enrolled or recently discharged from SAM treatment, were at least 18 years old, and spoke the lingua franca of the area, Bambara. Half of participants were recruited from children currently in treatment, and half were recruited from children discharged from treatment within the last three months, including those who had defaulted from treatment before reaching recovery. HCW’s were recruited from all personnel involved in delivering malnutrition treatment in the treatment sites of the six health areas.  After SSI’s, caregivers were recruited from the same health centers to participate in FGD’s of three to six participants. Caregivers were recruited such that approximately half had a child currently enrolled in SAM treatment and half had been discharged or defaulted in the past three months. Implementing HCW’s and supervisory HCWs were recruited based on their role delivering malnutrition treatment in the same selected sites.  **Phase Two**  Caregivers of children enrolled to the program were purposively sampled to participate in SSI’s based on the program registries, including caregivers who did not adhere to the scheduled program visits. The HCW’s primarily responsible for implementing the program were purposively sampled for individual interviews after a minimum of one month of implementing the program. SSI’s took place at the health site except in the case of abandoning the program, in which case the IDI was conducted at the caregiver’s home. |
| 12. Sample size | How many participants were in the study? | In Results section. |
| 13. Non-participation | How many people refused to participate or dropped out? Reasons? | We did not document participant refusal. |
| *Setting* |  |  |
| 14. Setting of data collection | Where was the data collected? e.g. home, clinic, workplace | SSI’s took place at the health site or caregiver’s home, depending on the caregiver’s preference and enrollment status.  FGD’s took place at the health site for implementing HCW’s, a monthly nutrition meeting for supervisory HCW’s, and a central community location for caregivers.  Direct observations took place at the health site during program activities. |
| 15. Presence of non-participants | Was anyone else present besides the participants and researchers? | No one partook in the research activity besides the participants and researchers, and a private setting was sought for all interviews and FGD’s. |
| 16. Description of sample | What are the important characteristics of the sample? e.g. demographic data, date | We did not collect individual level characteristics but report sample size and participants in data collection procedures and methods. |
| *Data collection* |  |  |
| 17. Interview guide | Were questions, prompts, guides provided by the authors? Was it pilot tested? | Interview guides were developed based on relevant literature and the expertise of the study team. The guides were pre-tested in team training (classroom test) and then during a field test to ensure clarity and accuracy (Data collection in Methods section). |
| 18. Repeat interviews | Were repeat interviews carried out? If yes, how many? | Some HCW’s who were interviewed in Phase One may have been interviewed in Phase Two (seeing the low caseload). We did not document repeat interviews. |
| 19. Audio/visual recording | Did the research use audio or visual recording to collect the data? | All interviews were audio-recorded following verbal consent (Data analysis in Methods section). |
| 20. Field notes | Were ﬁeld notes made during and/or after the interview or focus group? | Field notes and memos were written in every interview to capture insights (Data collection in Methods section). |
| 21. Duration | What was the duration of the inter views or focus group? | The interviews lasted up to one hour and participatory workshops up to 1 hour and 30 minutes (Data collection in Methods section). |
| 22. Data saturation | Was data saturation discussed? | Data saturation was assessed by organizing emerging results by research question during data collection. |
| 23. Transcripts returned | Were transcripts returned to participants for comment and/or correction? | Transcripts were not returned to participants; however, a reporting of findings of Phase One was included due to the iterative nature of the research in Phase Two of interview guides, and a presentation of the research findings was made to all HCW’s in the district at the end of the study. Due to the confidential nature of the research (no PII collected from caregivers), we did not track caregivers for a direct report back on findings. |
| **Domain 3: analysis and ﬁndings** |  |  |
| *Data analysis* |  |  |
| 24. Number of data coders | How many data coders coded the data? | Three independent coders (Data analysis in Methods section). A threshold of 0.8 was applied in Dedoose for a test of 15 codes on 44 excerpts.   - STK : 0.94 - INC : 0.83 |
| 25. Description of the coding tree | Did authors provide a description of the coding tree? | The coding tree was developed through Dedoose but summarized in the manuscript. |
| 26. Derivation of themes | Were themes identiﬁed in advance or derived from the data? | Themes were derived a priori from the interview guide and further derived from the data (inductive and deductive coding) (Data analysis in Methods section). |
| 27. Software | What software, if applicable, was used to manage the data? | Dedoose (Data analysis in Methods section). |
| 28. Participant checking | Did participants provide feedback on the ﬁndings? | Participants provided feedback on select findings of Phase One during Phase Two. |
| *Reporting* |  |  |
| 29. Quotations presented | Were participant quotations presented to illustrate the themes/ﬁndings? Was each quotation identiﬁed? e.g. participant number | Quotations were presented and participant profile provided while protecting confidentiality outlined in our informed consent forms (no geographic location identified to limit professional or personal risk). |
| 30. Data and ﬁndings consistent | Was there consistency between the data presented and the ﬁndings? | Consistency was checked (in Results section). |
| 31. Clarity of major themes | Were major themes clearly presented in the ﬁndings? | Major themes were clearly presented. |
| 32. Clarity of minor themes | Is there a description of diverse cases or discussion of minor themes? | Minors themes were described. |
