# Supplementary material for: Formative research on a post-discharge monitoring program using SQ-LNS to reduce acute malnutrition relapse risk in Mali : a qualitative study
Source: BMC Public Health. 2026 Jul 3;26:2296. doi: 10.1186/s12889-026-28378-x (PMC13445792; doi:10.1186/s12889-026-28378-x)
Supplement: Supplementary file 2 [file 12889_2026_28378_MOESM2_ESM.docx]

| **Code #** | **Brief Code Name** | **When to Use Code** |
| --- | --- | --- |
| **Section 1: Description of the health facility** | | |
| 1.1 | Demand fluctuations/ workload | - **Do not use this parent code** but instead use 1.1.1 – 1.1.3 below for coding when workload/demand fluctuates by unit of time at the health site are discussed. |
| 1.1.1 | Annual/ seasonal fluctuations | - Use this code when typical workload/demand fluctuates during certain periods of the year, for example based on seasonality (e.g. rainy versus dry seasons), on specific month periods (e.g. from December – June), etc. This could be either a health care worker or caregiver describing relevant annual or seasonal patterns. |
| 1.1.2 | Weekly fluctuations | - Use this child code when typical workload/demand fluctuates within during a typical week, for example busiest times during the weekend days, on specific market days, on Thursdays and Fridays, etc. This could be either a health care worker or caregiver describing relevant patterns within a given week. |
| 1.1.3 | Daily fluctuations | - Use this child code when typical workload/demand fluctuates within a given day of the week, for example busiest times in early morning, morning, late afternoon, etc. This could be either a health care worker or caregiver describing relevant patterns within a given day. |
| 1.2 | Workforce description | - **Do not use this parent code** but instead use 1.2.1 – 1.2.2 when the health worker describes the current workforce at their site. |
| 1.2.1 | Staffing | - Use this child code when the usual staff available at specific health center sites for malnutrition-related activities is described, including specific seasonal, weekly, and daily time availability. |
| 1.2.2 | Payment/ compensation/ financial motivation | - Use this child code when the payment, compensation, or financial motivation of staff is mentioned (including lack thereof). |
| 1.2.3 | Capacities | - Use this child code when the information in the text reflects the capacities/capabilities (e.g. level of education, knowledge and training, relevant skills and expertise) of the specific type of health worker being discussed, and in consideration of staffing for the relapse prevention-focused pilot/RCT. |
| **Section 2: Malnutrition treatment program (URENAS + URENAM)** | | |
| 2.1 | Treatment description | - Using this parent code is OK, particularly when usual malnutrition treatment procedures are described and specifically those that may apply to a post-treatment care program. This could include counseling, tools, and routine medications. This could also include treatment of SAM children as MAM when they reach MAM criteria (switching from red to yellow). **Do not use this code** when post-SAM treatment enrollment to MAM is mentioned (Use 3.1 instead) . |
| 2.1.1 | Factors influencing treatment | - Use this code if specific factors (barriers or enablers) of effective treatment are mentioned. |
| 2.2 | Screening description | - Using this parent code is OK, particularly when usual screening procedures (at the health site or in the community) are described and specifically those that may apply to a post-treatment care program. |
| 2.2.1 | Factors influencing screening | - Use this code if specific factors (barriers or enablers) of effective **malnutrition screening** are mentioned. |
| 2.3 | RUTF | - **Do not use this parent code** but instead use the child codes below for RUTF. |
| 2.3.1 | RUTF Stock(outs), monitoring | - Use this code when the health worker or caregiver describes RUTF stock and monitoring, including stockouts. This information should also capture factors influencing stockouts, including those modifiable and non-modifiable factors influencing stockouts in the context of programming. |
| 2.3.2 | RUTF Acceptability | - Use this code when caregiver/ child **acceptability** of RUTF is described, including information related to taste preferences, smell, packet colors/design/logo/name, and consistency of product, or caregiver sharing positive aspects of the RUTF (including child growth, other outcomes). This should be used for general impressions of RUTF and not utilization within the home or utilization/ consumption by the child. |
| 2.3.3 | RUTF Utilization | - Use this code when RUTF utilization in the home is described (behavioral factors). This code should capture both appropriate and inappropriate utilization patterns, including compliance patterns, barriers to compliance: sharing (intra- and inter-household sharing) used broadly (e.g. selling, bartering, consuming more/less than prescribed, others consuming, etc.), inability for child to finish sachet, and enablers: ease of preparation, ease of consumption, caregivers advice/tricks for ensuring child compliance, including strategies to reduce/avoid sharing practices. |
| 2.3.4 | Other (challenges/considerations) aspects of RUTF maybe relevant to SQLNS in programming | - Other challenges/ considerations related to RUTF that may be relevant to SQ-LNS in programming, but not captured in a previous RUTF code above. This can include storage and transit of RUTF. |
| 2.4 | RUSF/ MAM | - Use this code when RUSF or MAM programming is described, specifically and distinct from RUTF. This may be double coded with 2.3 code family above (RUTF). |
| **Section 3** | **Post-treatment monitoring** |  |
| 3.1 | Existing post-treatment care or monitoring | - Use this code when existing post-treatment care or monitoring is described. This could include measures at discharge from treatment such as counseling on best practices after treatment. This could also include enrollment to MAM post-SAM cure (per the 2017 protocol). During the confirmatory phase, this also could include descriptions of the program and/or comparisons to the program before. |
| 3.1.1 | Eligibility for post-treatment care with SQ-LNS | - Use this code if eligibility for post-treatment care during the confirmatory phase is described. |
| 3.1.2 | Exit from post-treatment care with SQ-LNS | - Use this code if exit procedures for post-treatment care during the confirmatory phase is described. |
| 3.2 | Perceptions of relapse | - **Do not use this parent code** but instead use the child codes below (3.2.1 and 3.2.2) for relapse-related descriptions. |
| 3.2.1 | Relapse burden | - Use this code when perceptions of relapse burden are described, for example what percentage of children admitted are relapsed children, or how. |
| 3.2.2 | Relapse causal factors | - Use this code for caregiver and health worker perceptions of causes of relapse are described, including those that may reflect any number of perceptions such as traditional beliefs, biomedical explanations, etc. |
| 3.3 | Specific actions for relapsed children | - Use this code if any specific actions for relapsed children are described by either health workers or caregivers |
| 3.4 | Reasons post-treatment monitoring not functional | - Use this code if reasons why the post-treatment monitoring program is not functional are either described or revealed. This code may include reasons related to upstream policies and guidelines, human or financial resources, limited political will, lack of organizational coordination, etc. This could also include lack of knowledge of the program by either the health care worker or the caregiver. |
| 3.5 | Suggested for post-treatment monitoring | - Use this code when design aspects of the program are discussed (where and when the program should be). Could be location, frequency, services, etc. Do not use this code during the confirmatory phase if suggestions for location, frequency, and other services are given when discussing placement of SQ-LNS; use 4.4 instead. |
| 3.6 | Resources needed for post-treatment monitoring | - Use this code when caregivers and health workers describe the resources needed for post-treatment monitoring including human resources (staff needed to deliver the program), operational resources (e.g. tools, supplies), financial resources, etc. |
| **Section 4** | **SQ-LNS** |  |
| 4.1 | Price | - Use this code for any mentions of SQ-LNS price considerations. This code will likely not refer to the nominal price but instead refer to opportunity costs related to accessing the SQ-LNS (e.g. time away from agricultural activities to travel to a facility for pick-up, financial costs for that travel to the health center, use of social capital to hitch a ride for accessing SQ-LNS at HC, husband permission needed for such travel, etc.). |
| 4.2 | Product | - Use this code for any mentions of a past experience with information that may reflect the SQ-LNS product itself, such as organoleptic properties of RUTFs that are liked/disliked (could be double coded with 2.3.2), colors/designs/logos of current supplements that may be considered for promoting the SQ-LNS, perceptions of product as medicine or food supplement, language written on the product including ingredients, questions related to whether it is halal vs haram, and other reflections on the product itself. |
| 4.2.1 | SQ-LNS Stock(outs), monitoring | - Use this code when the health worker or caregiver describes SQ-LNS stock and monitoring, including stockouts. This information should also capture factors influencing stockouts, including those modifiable and non-modifiable factors influencing stockouts in the context of programming. |
| 4.2.2 | Reported acceptability of SQ-LNS | - Use this code when health worker/ caregiver/ child **acceptability** of SQ-LNS is described, including information related to taste preferences, smell, packet colors/design/logo/name, and consistency of product, or caregiver sharing positive aspects of the SQ-LNS (including child growth, other outcomes). This should be used for general impressions of SQ-LNS and not utilization within the home or utilization/ consumption by the child. |
| 4.2.3 | Reported utilization of SQ-LNS | - Use this code for reported utilization of SQ-LNS during the confirmatory phase (including how it is given to children, how it is prepared, etc.) |
| 4.3 | Promotion | - **Do not use this parent code** but instead use the child codes below (4.3.1-4.3.3) |
| 4.3.1 | Trusted promoters | - Use this code for trusted promoters of SQ-LNS – meaning individuals and institutions trusted by caregivers for distributing and explaining a novel product. Use of this code may arise when the caregiver or health care worker is describing their experience with malnutrition treatment. |
| 4.3.2 | Channels | - Use this code for information related to channels for communication to promote SQ-LNS, including those that are social mobilization-based (e.g. child health days, sensitization events at the health site), media-based (e.g. print media such as brochures, community radio), and interpersonal-based (e.g. Care Groups, home visits by health workers using job aides). |
| 4.3.3 | Messages | - Use this code for information that may help inform the development of culturally-appropriate messages for promoting SQ-LNS, including possible local language names, words/phrases that caregivers use repeatedly and may be used for message creation, sensitization messages for RUTF that may be relevant to SQ-LNS (e.g. hygiene practices and safe storage of the product), caregiver aspirations for their children (e.g. ‘I want my child to become a teacher’) for message creation, core cultural values (e.g. mentions of Allah), and finally information to create cultural metaphors that may be used in messaging. During the confirmatory phase, this also includes messages shared with caregivers on best use of SQ-LNS. |
| 4.4 | Placement of SQ-LNS (program dimensions) | - Use this code when design aspects of SQ-LNS placement are discussed, including location, frequency, place, facility/community. During the confirmatory phase, this includes any reflections on or explanation of these dimensions. For specific recommendations on these dimensions, use the child codes below. |
| 4.4.1 | Specific recommendations on program location during confirmatory phase (Rec_location) | - Use this code when specific recommendations during the confirmatory phase are given regarding the program location/ site. |
| 4.4.2 | Specific recommendations on program frequency during confirmatory phase (Rec_frequency) | - Use this code when specific recommendations during the confirmatory phase are given regarding the program frequency. |
| 4.4.3 | Specific recommendations on program duration (Rec_duration) | - Use this code when specific recommendations during the confirmatory phase are given regarding the program duration. |
| 4.4.4 | Specific recommendations on program services (Rec_services) | - Use this code when specific recommendations during the confirmatory phase are given regarding the program services. |
| 4.5 | Resources needed to implement SQ-LNS program | - Use this code for resources (e.g. personnel, anthropometric tools, registries, participant cards, financials (payment or per-diems), transport, stationary, backpacks and name tag for health workers, support from government, etc.) needed to implement the SQ-LNS relapse-prevention program. This includes WHO is reliable and trusted to distribute the SQ-LNS (e.g., specific types of trusted health workers, community leaders, volunteer groups of women, etc.) |
| 4.6 | Other considerations related to SQ-LNS | - Use this code for other considerations related to SQ-LNS that do not fall into the above categories and may be emerging themes for additional code creation and/or just consideration in the subsequent pilot/RCT. |
| **Section 5** | **Information for pilot/RCT** |  |
| 5.1 | Miscellaneous information relevant to pilot/cRCT design | - Use this code for miscellaneous information that may be relevant to the pilot/ cRCT design and you are unsure where exactly it may fit into any of the categories above. During the pilot/confirmatory phase, this code may be used a lot for pieces of information that we need to take into consideration for the operational pilot that don’t fit in code 4.6 (Other considerations related to SQ-LNS). This code can be used on information that you are not entirely sure needs to be recorded but that you do see some potential value in retaining through the coding process. These codes may be then discussed more closely together among the analysis team during weekly meetings for consideration. |
| **Section 6** | **Content to review** |  |
| 6.1 | Review needed | - Use this code for acronyms, phrases and passages that do not make sense as translated and need to be reviewed. |
| 6.2 | Gem / Story or anecdote of note | - Any exemplar quote, story, or personal anecdote that should be used in publication to illustrate key point. |
| 6.3 | Emic terms/phrases | - Use this code to highlight individual words/phrases that cannot/were not translated from local language into French on the transcript. Many words/phrases cannot be translated from one language to another and these ‘emic’ terms are typically important to a cultural context and can be useful for message creation when promoting a product. - This code is the only one where we will agree to code less than a full sentence. |
